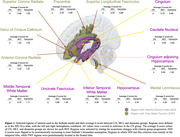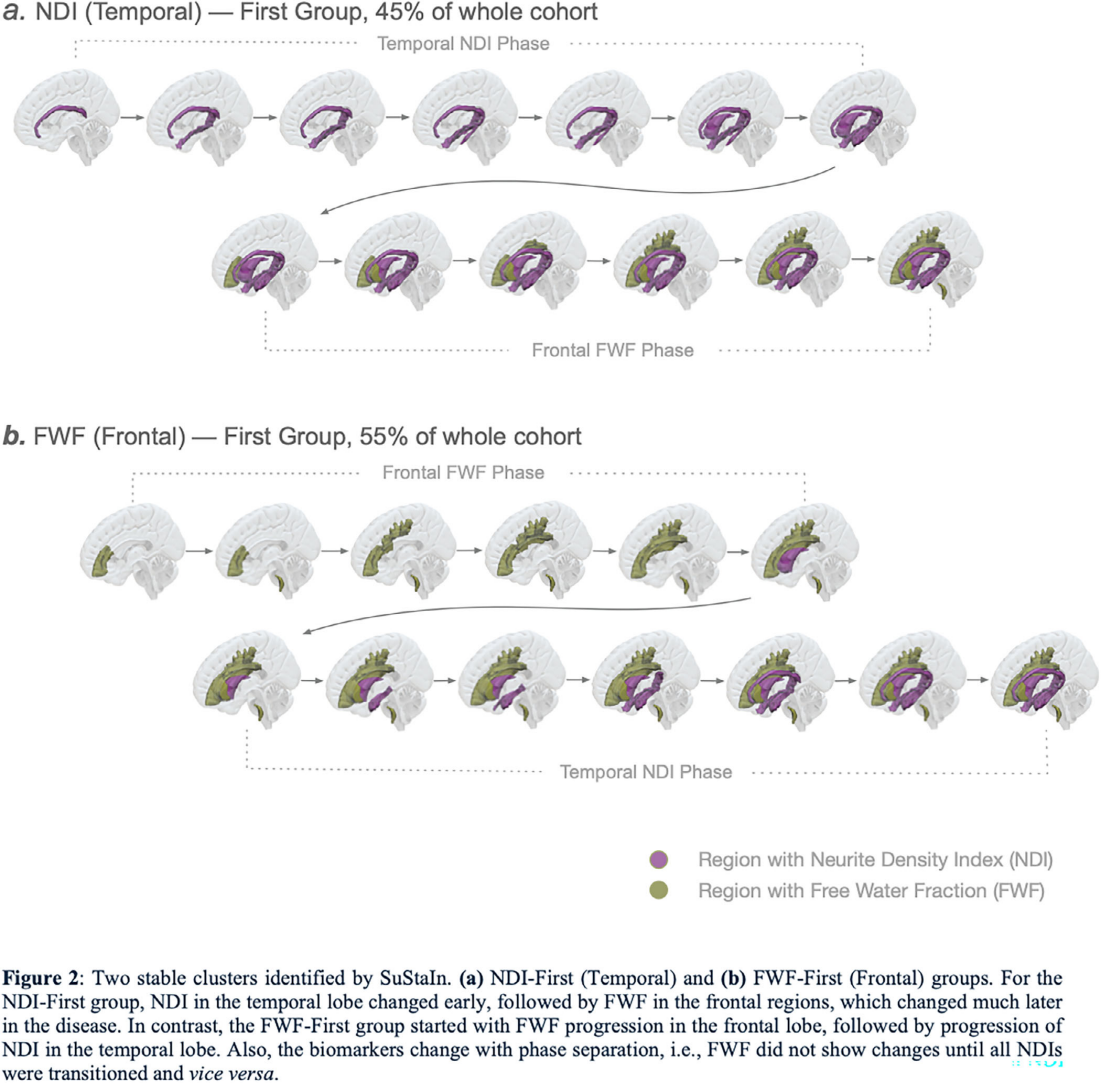# Distinct Spatial Patterns for White Matter Microstructure Changes in an Aging and Alzheimer’s Disease Cohort

**DOI:** 10.1002/alz.090927

**Published:** 2025-01-09

**Authors:** Jianqiao Tian, Sheelakumari Raghavan, Petrice M Cogswell, Robert I. Reid, Scott A. Przybelski, Christopher G. Schwarz, Jonathan Graff‐Radford, Val J. Lowe, Kejal Kantarci, David S. Knopman, Ronald C. Petersen, Clifford R. Jack, Prashanthi Vemuri

**Affiliations:** ^1^ Mayo Clinic, Rochester, MN USA; ^2^ Mayo Graduate School of Biomedical Sciences, Rochester, MN USA; ^3^ Department of Quantitative Health Sciences, Mayo Clinic, Rochester, MN USA; ^4^ Mayo Clinic, Radiology, Rochester, MN USA; ^5^ Department of Neurology, Mayo Clinic, Rochester, MN USA; ^6^ Mayo Clinic College of Medicine, Rochester, MN USA; ^7^ Department of Radiology, Mayo Clinic, Rochester, MN USA; ^8^ Mayo Clinic Alzheimer's Disease Research Center, Rochester, MN USA

## Abstract

**Background:**

White matter (WM) damage is seen with neurodegenerative and cerebrovascular pathologies and contributes to cognitive dysfunction. We hypothesized that there are multiple disease progression patterns in WM microstructural changes related to aging and dementia, and these can be identified using SuStaIn (data‐driven clustering algorithm for disease subtype and stage discovery) on multishell diffusion MRI (NODDI) data. NODDI provides information on cellular tissue architecture – neurite density index (NDI) measures packing density of axons and dendrites, and free water fraction (FWF) measures unrestricted water, e.g., interstitial and cerebrospinal fluid.

**Method:**

We identified 1136 participants (mean age=70 years, 51% male, 40% Aβ‐positive, 19% MCI/AD dementia) from the Mayo ADRC and Mayo Clinic Study of Aging. We analyzed NDI and FWF in 55 WM regions. We selected a subset of WM features that monotonically changed with clinical disease progression and then applied the Z‐score SuStaIn algorithm.

**Result:**

NDI in seven regions and FWF in six regions survived selection criteria (Figure 1). Noteworthy, the NDI regions were predominantly located in the temporal lobe, while FWF regions were primarily distributed in the frontal and subcortical regions. The SuStaIn algorithm discovered two stable clusters: the NDI‐First (Temporal) and FWF‐First (Frontal) groups (Figure 2). For the NDI‐First group, temporal lobe NDI changed early followed by frontal FWF. The FWF‐First group had the reverse order. There was clear phase separation based on which measure changed first. Stages of the NDI‐First group correlated better with meta‐ROI Tau‐PET SUVR and with MMSE than the FWF‐First group (p<0.01), suggesting that NDI‐First may be indicative of a primarily AD pathway.

**Conclusion:**

Given NDI changes are likely degenerative and FWF are suggested to be other processes such as neurodegeneration or inflammatory, the observed changes in the temporal and frontal regions may be due to distinct etiologies, i.e., neurodegenerative in the temporal lobes vs. vascular dysfunction often observed in the frontal lobes. The SuStaIn algorithm showed promise in discovering disease subtypes of WM microstructural changes. Further work is needed to understand the mechanistic basis of these observed changes and integrate WM changes for differential etiology identification and disease staging.